# Update on the Management of Pediatric Acute Osteomyelitis and Septic Arthritis

**DOI:** 10.3390/ijms17060855

**Published:** 2016-06-01

**Authors:** Luca Castellazzi, Marco Mantero, Susanna Esposito

**Affiliations:** 1 Pediatric Highly Intensive Care Unit, Department of Pathophysiology and Transplantation, Università degli Studi di Milano, Fondazione IRCCS Ca’ Granda Ospedale Maggiore Policlinico, 20122 Milan, Italy; lucacastellazzi@libero.it; 2 Respiratory Unit, Department of Pathophysiology and Transplantation, Università degli Studi di Milano, Fondazione IRCCS Ca’ Granda Ospedale Maggiore Policlinico, 20122 Milan, Italy; marco.mantero@unimi.it

**Keywords:** antibiotics, anti-infective therapy, osteoarticular infection, osteomyelitis, septic arthritis

## Abstract

Acute osteomyelitis and septic arthritis are two infections whose frequencies are increasing in pediatric patients. Acute osteomyelitis and septic arthritis need to be carefully assessed, diagnosed, and treated to avoid devastating sequelae. Traditionally, the treatment of acute osteoarticular infection in pediatrics was based on prolonged intravenous anti-infective therapy. However, results from clinical trials have suggested that in uncomplicated cases, a short course of a few days of parenteral antibiotics followed by oral therapy is safe and effective. The aim of this review is to provide clinicians an update on recent controversies and advances regarding the management of acute osteomyelitis and septic arthritis in children. In recent years, the emergence of bacterial species resistant to commonly used antibiotics that are particularly aggressive highlights the necessity for further research to optimize treatment approaches and to develop new molecules able to fight the war against acute osteoarticular infection in pediatric patients.

## 1. Introduction

Acute osteomyelitis and septic arthritis represent two inflammatory diseases that affect bone and synovial joints and are both primarily caused by bacterial infection [1,2,3]. These two diseases can occur alone or in combination. Traditionally, their incidence is approximately eight cases per 100,000 children per year, with high prevalence in those aged ≤5 years. However, in recent years, an increase in their incidence has been observed. There is a higher incidence in males, most likely because they are more physically active, which predisposes them to repeated microtrauma [1,2,3]. Hips, knees, and ankles are the most frequently involved joints [4,5,6].

The pathogenesis of acute osteomyelitis and septic arthritis may occur with three different mechanisms: the pathogen’s dissemination via the blood, extension by contiguity, or penetration of the infectious agent [6]. The first mechanism is the most frequently described in children.

Acute osteomyelitis and septic arthritis, although not so frequent, should not be underestimated because they could be associated with sepsis and with sequelae such as joint destruction, growth failure, and death of the patient if they are not correctly treated [7,8]. Therefore, early diagnosis and initiation of proper treatment are essential to obtain a better outcome and avoid sequelae. Furthermore, the emergence of bacterial strains particularly virulent and resistant to antibiotics has made the treatment of these conditions more difficult than in the past and has led to the need to have new antibiotic molecules available to deal with these emergencies [7,9]. However, there is no consensus in the literature regarding the best antibiotic therapy to be administered, the mode of administration, and the duration of therapy. These topics are still the subject of an ongoing debate among experts. The aim of this review is to provide clinicians an update on recent controversies and advances regarding the management of acute osteomyelitis and septic arthritis in children.

## 2. Controversies in Management

Antibiotic therapy is the gold standard for treating acute osteomyelitis and septic arthritis. The antibiotic is initially chosen on an empirical basis to cover the most frequent pathogens responsible for these conditions based on the age of the child and is then driven on the basis of the antibiograms obtained from the cultural investigations performed before starting antimicrobial therapy (such as blood culture or, when available, intra-articular liquid or bone fragment) [10]. However, there is an urgent need for treating all the infants and children who are admitted with these diseases due to their severe clinical conditions and risks of complications. Unfortunately, in several cases, the blood culture is negative and the other invasive procedures, different from what is done in adults, are not performed in the first years of life. Therefore, it is important to know the epidemiology reported in the literature for different age groups to help choose an appropriate empirical treatment.

*Staphylococcus aureus* is definitely the most frequent pathogen responsible for osteomyelitis and septic arthritis in any age group, mainly methicillin-sensitive strains (MSSA), and it is responsible for up to 70%–90% of confirmed cases [11]. As noted in the adult population, there has also been an increase in cases of osteoarticular infection due to methicillin-resistant strains of *S. aureus* (MRSA) in pediatric patients that should consequently be considered when choosing an empirical antimicrobial treatment [7,12].

In children less than two months of age, *Streptococcus agalactiae* and other Gram-negative organisms are recognized as potential pathogens. However, in children between two and five years of age, *Streptococcus pyogenes* and *Streptococcus pneumoniae* should be considered [13]. *Haemophilus influenzae* type b was considered a common cause of acute osteoarticular infections in children [14]. Fortunately, after the introduction of large-scale vaccination programs, the number of cases of acute osteomyelitis and septic arthritis due to *H. influenzae* type b has drastically decreased.

An emerging pathogen in the etiology of pediatric acute osteoarticular infection is *Kingella kingae*. It is a common commensal of the oropharynx of children that affects children between six months and four years of life in particular, and it is difficult to isolate [15]. Using aerobic blood culture vials or real-time polymerase chain reaction (PCR), the bacterium can be more easily identified. Some authors showed that *K. kingae* appeared as the most frequent etiologic agent of acute osteoarticular infections in children aged less than four years, excluding the neonatal period [16,17].

Rare causes of osteomyelitis in pediatric patients are *Mycobacterium tuberculosis*, *Bartonella henselae*, and fungi (*i.e.*, *Histoplasma* spp. and *Cryptococcus* spp.) [4,5,6]. These pathogens should be considered mainly in immunocompromised children.

Given the distribution of pathogens in the different age groups, in neonates, empirical treatment should include oxacillin with the addition of gentamicin [18,19]. In children older than three months, to cover MSSA, *S. pyogenes*, and *K. kingae*, antistaphylococcal penicillin such as nafcillin or oxacillin or a first-generation cephalosporin such as cefazolin should be used [18]. However, in the case of a high suspicion of MRSA infection (*i.e.*, countries with a prevalence of MRSA ≥10%, patients previously hospitalized in the intensive care unit, immunocompromised patients), antibiotic coverage against this pathogen should be recommended. In these cases, a first choice may be the use of clindamycin, an antibiotic belonging to the class of lincosamides, the effectiveness of which has been shown [20]. If the percentage of local resistance to clindamycin is greater than 10%, the drug of first choice is vancomycin [21,22]. However, this glycopeptide has the disadvantage of requiring intravenous administration and therapeutic drug monitoring for possible toxic effects. In cases of MRSA that are unresponsive to clindamycin and vancomycin, an alternative option is linezolid [8,11,23]. Linezolid belongs to the class of oxazolidinones, and it is effective against infections caused by Gram-positive bacteria, such as MRSA, coagulase-negative staphylococci, glycopeptide-resistant enterococci, and penicillin-resistant *Streptococcus pneumoniae*. Linezolid also has the advantage of having very good absorption even orally. Additionally, various studies have established the required dosages in neonatal and pediatric patients; however, it is expensive and, in cases of protracted therapies, may be associated with the development of hematological abnormalities as well as optical and peripheral neuropathies [19,24].

Even daptomycin, a lipopeptide antibiotic, may be considered a useful antibiotic in the case of multi-resistant Gram-positive bacteria. However, further studies in neonatal and pediatric patients are required to validate the use of this antibiotic in children [25].

Trimethoprim-sulfamethoxazol has been successfully used for the treatment of skin and soft tissue infections due to MRSA [26]. A small retrospective study by Messina *et al.* showed that in the case of pediatric osteomyelitis due to MRSA, trimethoprim-sulfamethoxazol represents an effective treatment option [27]. However, larger clinical trials are needed to confirm this therapeutic choice [28].

In recent years, acute osteoarticular infections due to *S. aureus* strains producing Panton-Valentine leukocidin have been reported, although not so frequently in Europe [29,30,31,32,33]. Panton-Valentine leukocidin (PVL) is a toxin that causes tissue necrosis and destruction of the neutrophils, thereby facilitating the extension of the infection, and children affected by this pathogen have more aggressive clinical features [21,22,30,34,35,36]. The risk of complications such as subperiosteal abscesses, pyomyositis, necrotizing fasciitis, and orthopedic sequelae is high [37]. Therefore, cases of bone or joint infection caused by *S. aureus* strains producing PVL require more aggressive and prolonged antimicrobial treatment and often require repeated surgical debridement [32,38]. The use of fluoxacillin, clindamycin or linezolid is recommended in these cases, with daptomycin considered as a second-line antibiotic. Daptomycin was successfully used in a recent case report of a child with PVL-positive staphylococcal osteomyelitis [39].

*Salmonella* spp. is a frequent cause of acute osteoarticular infection in developing countries and in patients with sickle cell disease [40,41,42]. For this type of infection, a third-generation cephalosporin such as cefotaxime or ceftriaxone or a fluoroquinolone should be considered [43].

Finally, *Candida* spp. is another pathogen that may be identified from some cases of acute osteomyelitis (mainly spondylodiscitis) which requires a prolonged antifungal treatment and surgical debridement [44].

However, the identification of the pathogen responsible for the acute osteoarticular infection requires time due to the slow growth of some etiologic agents. Moreover, invasive procedures that require the patient’s sedation are limited in pediatric patients for ethical reasons. According to various studies, it seems possible in anywhere from 30% to 70% of cases, and the severity of the clinical picture as well as the patient’s age are key aspects for the selection of antibiotic therapy [22].

## 3. The Duration of Anti-Infective Treatment

Acute osteomyelitis and septic arthritis require prompt anti-infective treatment, starting with intravenous antibiotics and then transitioning to oral antibiotics to avoid complications. However, there is an absence of consensus regarding antibiotic duration and when to change to oral treatment.

Traditionally, children with acute osteomyelitis and septic arthritis receive intravenous antibiotic therapy for several weeks, then switch to oral therapy when healing has almost been achieved [43]. However, the long duration of parenteral therapy was associated with prolonged hospitalization, high cost, and sometimes the need for central venous access. Some centers start the peripheral intravenous therapy for some days during hospitalization and then insert a central venous catheter to provide four to six weeks of parenteral therapy at home [45].

Some authors have suggested the possibility of reducing the duration of intravenous antibiotic therapy to only a few days and then continuing the therapy orally [3,33,46,47,48,49,50,51,52]. In a prospective randomized study performed by Peltola *et al.*, pediatric patients with acute osteomyelitis caused primarily by MSSA strains treated with oral antibiotics (clindamycin or a first-generation cephalosporin) for 20 days attained the same efficacy as patients treated for 30 days after an initial intravenous treatment of two to four days in both groups [48]. Similar results were obtained by Jagodzinski *et al.* in 70 children between two weeks and 14 years of age suffering from acute osteomyelitis or septic arthritis who were treated for three to five days with intravenous therapy at high doses and then later with an oral therapy for three weeks [49]. However, in the case of MRSA or PVL *S. aureus*, four to six weeks of treatment are recommended [23].

Also, it was demonstrated that two to three weeks of large doses of well-absorbed antibiotics started intravenously and only one joint aspiration appear to be sufficient for the treatment of septic arthritis in children [53]. The oral administration of antibiotics exposes the patient to a lower risk of complications associated with prolonged antibiotic parenteral administration at home and results in fewer visits to the emergency department and lower rehospitalization rates [54,55].

Undoubtedly, the child should not present severe complications, must be able to assume the antibiotic therapy orally, and should tolerate the drug; the antibiotic should also have high oral bioavailability and reach adequate concentrations at the site of the infection [8]. Finally, the oral antibiotic must have the same degree of antibacterial coverage as the parenteral drug [19].

The transition from intravenous antibiotic therapy to oral therapy can also be guided by other factors. In particular, an improvement in the general condition of the child, stable apyrexia, and a significant reduction in C-reactive protein (CRP) should be present. Recently, Chou *et al.* observed that a 50% reduction in CRP associated with a clinical improvement of the patient can be used for the transition to oral antimicrobial therapy in acute bacterial osteoarticular infection [56]. Similar results were obtained in a retrospective study by Pääkkönen *et al.* in which patients affected by uncomplicated osteomyelitis of the calcaneus not caused by MRSA were analyzed [57]. Arnold *et al.* [58] showed 99.5% success in 194 pediatric patients with acute bacterial osteomyelitis who were switched to oral therapy as a result of an improvement of the clinical picture and CRP < 2 mg/dL. Only one patient experienced treatment failure, but this was due to a retained infected bone fragment in the hip joint [58]. Finally, a CRP value of less than 2 mg/dL appeared to be a useful indicator for suspending antibiotic therapy in acute bacterial osteoarticular infection [58].

However, complicated cases of acute osteoarticular infections, including those that occur at neonatal age or in immunocompromised children as well as cases due to *Salmonella* spp. or MRSA, require longer treatment [50,59]. Interestingly, Jagodzinski *et al.* demonstrated that initial CRP levels higher than 10 mg/dL and a temperature above 38.4 °C should be considered predictors of the need for a prolonged course of intravenous antibiotic administration [49]. In cases where the values of CRP are persistently elevated despite antibiotic therapy, a complication should be suspected and antibiotic therapy should be modified or prolonged [19].

In conclusion, a short course of intravenous antibiotic treatment followed by an oral antimicrobial therapy for two to three weeks in septic arthritis and three weeks in osteomyelitis appears to be safe and effective in cases of uncomplicated osteoarticular infection. In the other cases, intravenous therapy should be prolonged for at least three weeks in septic arthritis and four to six weeks in osteomyelitis. However, the duration of anti-infective treatment should always rely on clinical judgment, objective diagnostic data, and oral bioavailability of the different antibiotics.

## 4. Other Drugs

In the case of septic arthritis, two clinical trials studied the effects of dexamethasone on antibiotic therapy. In both of these double-blind, placebo-controlled studies, it was observed that four days of intravenous dexamethasone together with antibiotic therapy resulted in a shorter duration of symptomatology as well as a minor residual dysfunction of the joint at the end of antibiotic therapy and during follow-up [60,61].

These results were recently confirmed in a large retrospective study by Fogel *et al.* [62] who analyzed 116 pediatric patients with septic arthritis, 90 of whom were treated with antibiotics alone and 26 of whom were treated with antibiotics in addition to intravenous dexamethasone for a few days. The latter group experienced a shorter duration of fever (mean 2.3 *vs.* 3.9 days, *p* = 0.002), a more rapid clinical improvement (mean 6.3 *vs.* 10.0 days to no pain/limitation, *p* < 0.001), a more rapid decrease in CRP levels to <1 mg/dL (mean 5.3 *vs.* 8.4 days, *p* = 0.002), a shorter duration of parenteral antibiotic treatment (mean 7.1 *vs.* 11.4 days, *p* < 0.001), and a shorter hospital stay (mean 8.0 *vs.* 10.7 days, *p* = 0.004) [62].

However, although these studies suggest that treatment with corticosteroids is associated with a better outcome, more studies are needed to confirm these results [63].

Non-steroidal anti-inflammatory agents (NSAIDs) should be used to reduce pain [1].

Furthermore, in adult patients with orthopedic implants it has been shown that biofilm may cause failure of antibiotic treatment [64,65]. *In vitro* findings have suggested that local delivery of antimicrobials may be an effective strategy for the prevention and/or treatment of open fractures where biofilms might develop [66,67].

## 5. Surgery

Together with antibiotics, surgery plays a key role in the treatment of acute osteomyelitis and septic arthritis in children [3].

First, surgery makes it possible to obtain biological samples that are useful for identifying the etiologic agent and then guiding the selection of the correct antibiotic to use for treatment. Moreover, in cases with joint involvement, surgical drainage of the joint reduces the risk of complications such as avascular necrosis of the bone and permanent cartilage damage due to increased intra-articular pressure and results in a better outcome [50]. Furthermore, arthroscopic lavage was found to be safe and effective for the treatment of septic arthritis in very young children [68]. In addition, surgery alters the process of bone necrosis, which reduces the vasculature and therefore the penetration of the antibiotic at the site of infection, removes the demineralized bone, and cleans the surrounding soft tissue, thereby reducing the bacterial load [38].

However, in acute uncomplicated cases of osteoarticular infection, surgery may not be necessary [22]. It should be considered in patients who do not respond to antibiotic treatment for the suspicion of an underlying complication. Acute osteoarticular infections caused by MRSA or *S. aureus* producing PVL often require more surgical sessions as these bacteria are associated with a more aggressive clinical course [50].

## 6. Conclusions

Acute osteomyelitis and septic arthritis are two infections whose frequencies are increasing in pediatric patients. Acute osteomyelitis and septic arthritis need to be carefully assessed, diagnosed, and treated to avoid devastating sequelae.

It is currently accepted that initial treatment of acute osteomyelitis and septic arthritis involves empirical antibiotic therapy that should cover the pathogens potentially responsible for these infections based on age groups. However, there is no consensus on which molecule(s) should be used in various countries. This lack of consensus is mostly due to the different antibiotic-resistant microorganisms in the different regions of the world. Consequently, the choice of antibiotic should be individualized to cover the microbial profiles of the different regions, as well as considering the patient’s age and clinical status.

In cases of acute uncomplicated osteomyelitis and septic arthritis, antibiotic treatment can be provided intravenously for a short period of a few days and then orally until the end of the disease. The improvement in the clinical status and the inflammatory markers can be used by clinicians to decide on the timing of the switch to oral therapy. The latter should only be administered if the patient is capable of taking it adequately and only if it has the same spectrum of coverage as the antibiotic used parenterally.

In the last few years, the emergence of bacterial strains particularly virulent and resistant to antibiotics has been observed. These pathogens are associated with highly aggressive and dangerous clinical features. Consequently, in these cases, as well as in the neonates and in patients with chronic underlying disease, it is extremely important to obtain as much reliable sample as possible for microbiological examination and to start a prompt, aggressive, prolonged intravenous treatment.

However, several gaps in the management of acute osteoarticular infections remain. There are relatively few studies of the diagnostic methods that should be performed for the identification of the different pathogens and the most appropriate parameters to be used in children to switch from parenteral to oral antibiotic therapy in relation to the etiology. Consequently, further research should focus on these issues to optimize the duration of intravenous and oral anti-infectives. Moreover, further studies of the quantitative impact of MRSA and *S. aureus* PVL-producing strains in these infections as well as on the optimal treatment in terms of the molecule of choice and the duration of antibiotic administration are mandatory. In addition, limited data on the efficacy and safety of corticosteroids in patients with acute osteoarticular infection are available, and further studies on this issue should be useful. Furthermore, the role of biofilm in treatment failures in pediatric patients should be evaluated. Finally, the emergence of bacterial strains resistant to antibiotics makes it imperative to focus the research on the search for new molecules that are capable of adequately penetrating bones and joints to overcome this problem.
